# Biomass composition: the “elephant in the room” of metabolic modelling

**DOI:** 10.1007/s11306-015-0819-2

**Published:** 2015-06-11

**Authors:** Duygu Dikicioglu, Betul Kırdar, Stephen G. Oliver

**Affiliations:** 1 Cambridge Systems Biology Centre & Department of Biochemistry, University of Cambridge, Cambridge, CB2 1GA UK; 2 Department of Chemical Engineering, Bogazici University, Istanbul, Turkey

**Keywords:** Biomass composition, Flux balance analysis, Macronutrient limitation, Metabolic modelling, Energy cost

## Abstract

Genome-scale stoichiometric models, constrained to optimise biomass production are often used to predict mutant phenotypes. However, for *Saccharomyces cerevisiae*, the representation of biomass in its metabolic model has hardly changed in over a decade, despite major advances in analytical technologies. Here, we use the stoichiometric model of the yeast metabolic network to show that its ability to predict mutant phenotypes is particularly poor for genes encoding enzymes involved in energy generation. We then identify apparently inefficient energy-generating pathways in the model and demonstrate that the network suffers from the high energy burden associated with the generation of biomass. This is tightly connected to the availability of phosphate since this macronutrient links energy generation and structural biomass components. Variations in yeast’s biomass composition, within experimentally-determined bounds, demonstrated that flux distributions are very sensitive to such changes and to the identity of the growth-limiting nutrient. The predictive accuracy of the yeast metabolic model is, therefore, compromised by its failure to represent biomass composition in an accurate and context-dependent manner.

**Electronic supplementary material:**

The online version of this article (doi:10.1007/s11306-015-0819-2) contains supplementary material, which is available to authorized users.

## Introduction

When the entire metabolic network is viewed as a multi-enzyme system, small changes in enzyme concentrations have been found not to elicit profound changes in the overall metabolic flux, demonstrating the robustness of metabolic pathways (Matias Rodrigues and Wagner 2009). This non-linearity in the relationship between enzyme activity and metabolic flux indicated that the effect of a small change in enzyme activity produced a major change in the flux when the enzyme’s activity was low. This is explained, by metabolic control theory (Kacser and Burns 1981), as indicating that most enzymes have a negligible effect on the flux through a pathway unless their activity level becomes limiting. Metabolic pathways themselves evolved through natural selection in order to be robust to both environmental and genetic perturbations, including mutations (Barve and Wagner 2013; Crow and Simmons 1983; Matias Rodrigues and Wagner 2009; Mayo and Burger 1997; Snitkin et al. 2008).

Systematic gene deletion studies conducted in the yeast *Saccharomyces cerevisiae* showed that <20 % of the organism’s protein-encoding genes are essential for viability as determined by growth on a rich, glucose-containing medium (Giaever et al. 2002; Winzeler et al. 1999). Moreover, many deletion mutants were able to grow at rates equal or close to those of the wild type under a number of defined environmental conditions, indicating that the biological networks of the organism provides a robustness in its internal wiring that buffers against genetic variations (Thatcher et al. 1998). This emphasized an important property of biological networks that had been recognised previously, namely their resistance to attack at single node (Albert et al. 2000; Deutschbauer et al. 2005).

Flux balance analysis (FBA) was used to predict metabolic phenotypes under different conditions, such as substrate and oxygen availability, by simply constraining the appropriate fluxes to predict a particular flux distribution using linear optimization (Dikicioglu et al. 2008; Gombert and Nielsen 2000; Kauffman et al. 2003). It was previously reported that living systems might change their biological objective when a physiological change was imposed on them (Almaas et al. 2005). Thus, our understanding of such changes limits the ability of FBA to correctly describe the system. Another factor limiting our ability to describe metabolism using FBA is the accuracy with which the composition of biomass is represented in the model. Moreover, accurate biomass representations enhance our capability to infer suitable cellular objectives in order to improve the predictive capability of metabolic flux analysis (Almaas et al. 2005). This is because the composition of yeast biomass varies in response to the physiological challenges to which the cells are exposed [reviewed in (Verduyn et al. 1991)]. Previous studies highlighted the significance of the experimental determination of biomass composition in flux calculations in metabolic models, reporting the need for precise measurements and careful validation in order to determine flux-derived parameters (Lange and Heijnen 2001; Wang and Stephanopoulos 1983). Although these workers provided clear outlines of the experimental and statistical protocols to be used, they failed to provide a comprehensive analysis of yeast biomass composition.

In this study, we have investigated, in silico, the effect of nutrient availability and biomass composition on the distribution of predicted fluxes in the metabolic network of *S. cerevisiae*. We first focused on high-flux pathways to determine whether the magnitudes of the fluxes were indicative of a high metabolic burden associated with those pathways or whether they were indicative of misleading representations of the metabolic network of yeast in the most recent version of its genome-scale model. We next investigated how variations in macronutrient availability and biomass composition affected the predictive abilities of this model.

## Materials and methods

### Experimental methods

The wild-type *S. cerevisiae* diploid strain BY4743 [*MAT* ***a***/*MATα* *his3Δ*/*his3Δ* *leu2Δ*/*leu2Δ* *LYS2*/*lys2Δ* *MET15*/*met15Δ* *ura3Δ*/*ura3Δ*; (Brachmann et al. 1998)] was cultivated in 2L fermenters (Sartorius Stedim Biotech, Germany) with 1L working volume under aerobic conditions (0.1 vvm, 800 rpm, ≥80 % dO_2_ saturation) in synthetic defined medium (Baganz et al. 1997); all chemicals were purchased from Merck KGaA, Germany and Sigma-Aldrich, USA) operated in batch mode. Temperature and pH were controlled at 30 °C and 4.5, respectively. Fermentations were carried out in triplicate with samples taken at hourly intervals during the exponential growth phase to determine glucose and ammonium utilization as well as ethanol and glycerol production. The dry weight was determined gravimetrically. Extracellular metabolite concentrations were determined enzymatically [R-Biopharm (Germany) Yellow Line Enzymatic BioAnalysis and Food Analysis kits (Cat no: 10 139 041 035 Sucrose/d-Glucose, 10 148 270 035 Glycerol, 10 176 290 035 Ethanol, 11 112 821 035 Ammonia)] as described by the manufacturer.

### Metabolic modelling

The Yeast 7.00 stoichiometric model of the *S. cerevisiae* metabolic network (Aung et al. 2013) was employed in FBA. In order to make sure that the results were not specific to a particular version of the model, and that they were persistent and intrinsic to any yeast stoichiometric metabolic model, the analysis was repeated using the first genome-scale model of yeast [iFF708 (Famili et al. 2003)] and with Yeast 4.00 [an earlier version of the current model (Dobson et al. 2010)]. The maximization of biomass production with absolute flux minimization was used as the objective function. The simulations were carried out by running the COBRA Toolbox (v2.0.3) under MATLAB R2012b (8.0.0.783, Mathworks, USA) with SBML Toolbox v4.0.1 and libSBML library v5.0.0b0 using standard linear optimization techniques (GLPK toolbox). FAME was also employed as the flux analysis modelling and visualization environment (Boele et al. 2012). Mutant flux distributions were also investigated employing the MoMA algorithm as described in (Segrè et al. 2002). Flux variability analysis and MoMA analyses were carried out using the same set-up as that used for the FBA. Medium compositions and product concentrations were introduced to the system as bounding constraints whenever available. In the absence of experimental measurements, the complex medium was simulated with 2 % glucose setting the constraints for its uptake; the synthetic defined minimal medium was simulated by setting the constraints for the components of the footprinting medium for the uptake fluxes (FPM) (Chiu and Segrè 2008). A macronutrient (glucose, ammonium, sulphate or phosphate) was considered as limiting at 10 % of its concentration in the original medium recipe and the system was thus constrained to maximally uptake the specified amount from the extracellular environment. Balanced growth with a specific growth rate of 0.1 h^−1^ was used unless otherwise specified. The coefficient of every constituent in the model biomass equation was varied within a fourfold range of its documented value to explore the limits of the available solution space. This range was expanded or contracted as needed for the sensitivity analysis.

### Data analysis

The multiplicative model of epistasis (μ_AB_ − μ_A_ × μ_B_) was used to determine the synthetically lethal gene pairs where no growth was represented as μ < 0.0001 h^−1^. Only synthetically lethal interactions, which occur at a frequency of ca. 1 % across all pairwise combinations of genes in the *S. cerevisiae* genome; (Boone et al. 2007), were investigated, and not all cases of epistasis between gene pairs.

Princeton GO Tools was used for the Ontology definitions (accessed in 03/2014) and the Generic Gene Ontology (GO) Term Finder was employed to conduct the GO term enrichment analysis using hypergeometric distribution for multiple hypothesis testing (Boyle et al. 2004).

The flux distribution data were analysed using standard statistical techniques available in Microsoft Excel and MATLAB R2012b with Statistics Toolbox (8.0.0.783, Mathworks, USA). 2-tailed dependent Student’s *t* test was used for evaluation of significance for paired samples. Principal components analysis (PCA) and partial least squares regression (PLSR) were conducted on z-score normalized data centred around 0 (µ) and scaled by 1 (σ). Singular value decomposition was used in the PCA analysis. PLSR was conducted on 1740 predictor variables with 73 observations (SBC + 72 BRs) in each case of macronutrient limitation. Pearson correlation coefficient was used for determining the degree of linear dependence between the fluxes.

## Results and discussion

### Quasi-steady-state flux balance analysis and the efficiency with which energy-associated pathways are utilised

The stoichiometric model of the *S. cerevisiae* metabolic network (Aung et al. 2013) was constrained using growth, substrate consumption, and by-product formation rates determined for wild-type yeast cells, which were grown at an apparently constant rate in a chemically defined medium in carefully controlled batch fermentations. The in silico distribution of metabolic fluxes was observed to be in good agreement with the empirical observations during early-to-mid exponential phase using the optimization of biomass production with the minimization of absolute fluxes as the objective function, also taking the alternative optima into consideration through flux variability analysis (ESM1, ESM1 and ESM3).

A total of 460 enzyme-encoding genes (whose products determine 19 % of all fluxes) were associated with reactions with non-zero fluxes. The fluxes, whose absolute values were determined to be less than 1 % of the maximum absolute reaction flux in magnitude, were considered as inconsequential and were eliminated from this analysis. This left a set of fluxes associated with 121 unique enzyme-encoding genes, which we call the “highly-elevated flux sub-network” (HFS). The HFS was enriched for enzyme reactions that have low variability as given by the flux variability analysis and ca. 3 % of yeast’s metabolic reactions were reported to be always active under different simulated growth conditions while the remaining reactions are conditionally active and respond to specific environmental changes, defined as the flux-based plasticity (Almaas et al. 2005).

HFS genes were determined to be significantly enriched for GO process term “generation of precursor metabolites and energy” (p value = 9.66E−43). We further identified a subset of reactions with “extremely elevated fluxes”, for which the magnitude of the computed absolute fluxes was greater than 10 % of the value determined for the maximum absolute reaction flux. These reactions are catalysed by enzymes specified by 31 unique metabolic genes that are significantly associated with ATP synthesis, coupled proton transport (p value = 5.94E−36), and amino-acid catabolic process to alcohol via the Ehrlich pathway (p value = 5.02E−03) (ESM4, ESM5 and ESM6). It was previously suggested that the overall intracellular flux distribution could be minimized since microorganisms have evolved to maximize enzymatic efficiency in order achieve rapid growth rates (Bonarius et al. 1996). Despite the measures taken to reduce the absolute values of the fluxes distributed within the metabolic network, some sub-sets of the network were observed to carry a higher load of the total metabolic burden, this being associated with higher net fluxes through these pathways. Networks in which reactions mediated by enzymes that are products of HFS genes were then investigated for their growth phenotypes since we would expect any reduced fitness to be reflected in reduced growth rate (Schulz zur Wiesch et al. 2010).

### The predictive capability of the model is poor for genes encoding enzymes of the high flux sub-network

HFS genes were used as queries for the simulation of the viability of null mutants, where the predicted and the documented growth phenotypes were assigned into Boolean classes of 1 or 0, indicating the presence or absence of growth. The null hypothesis (H_0_) to be tested was that the strains predicted to be inviable in the model-based simulations would indeed prove to be inviable under the conditions for which empirical data were available [complex medium with 2 % glucose, limited oxygen availability; downloaded from the SGD (Cherry et al. 2012) database (http://downloads.yeastgenome.org/curation/literature/phenotype_data.tab (03/2014)]. The viable/lethal phenotype could correctly be predicted for 92 % of the query enzymes (Table 1; ESM7). A viable phenotype for a strain bearing a deletion in an HFS gene (96 %) could be more accurately predicted than those in the entire genome-scale metabolic network (82 %) (CN). In contrast, the prediction of an inviable phenotype for deletion mutants of genes encoding enzymes in the HFS could not be successfully predicted (45 %). This performance was considerably poorer than that for predictions carried out on CN (78 %). Furthermore, essential genes were under-represented in the HFS (8 % of the genes) in comparison to that in the CN (28 %) (Table 1; ESM7). Use of minimization of metabolic adjustment (MoMA), which is proposed specifically for simulating mutant flux distributions, yielded similarly inaccurate predictions of essentiality.

**Table 1:** A comparison of the predictive ability of HFS and CN

	Viability analysis		Synthetic lethality (SL) analysis	
	CN	HFS	CN	HFS
No. of genes/gene pairs	911	118	281,625 pairs	6441 pairs
Essential gene/SL fraction	28 %	8 %	0.08 %	2.53 %
No. of TP	619 (68 %)	103 (87 %)	281,105 (278,087)	6255 (6126)
No. of FP (Type I error)	132 (14 %)	4 (3 %)	281 (3299)	23 (152)
No. of TN	124 (14 %)	5 (4 %)	33 (40)	2 (7)
No. of FN (Type II error)	35 (4 %)	6 (5 %)	206 (199)	161 (156)
Sensitivity^a^	95 %	94 %	99.92 % (99.92 %)	97.49 % (97.52 %)
Specificity^a^	48 %	56 %	10.51 % (1.20 %)	8.00 % (4.40 %)
PPV^a^	82 %	96 %	99.90 % (98.83 %)	99.63 % (97.58 %)
NPV^a^	78 %	45 %	13.80 % (16.74 %)	1.23 % (4.29 %)
Predictive success^a^	82 %	92 %	99.83 % (98.76 %)	97.12 % (95.22 %)

^a^PPV = TP/(TP + FP), NPV = TN/(TN + FN), sensitivity = TP/(TP + FN), specificity = TN/(TN + FP), predictive success = (TP + TN)/no of genes

^b^The comparison of the predicted values with documented synthetically sick or lethal (SSL) pairs is provided in parentheses in the last two columns

Most of the HFS genes were found to be involved in energy-associated processes and were tightly linked with central carbon metabolism. The fact that essential genes were under-represented in this gene set is indicative of genetic redundancy and the fact that isozymes exist for many of these fundamental reactions, thus providing alternative routes and increasing the robustness of this core network. It may also be the case that the enzymes encoded by these essential genes were more optimized for their metabolic function and thus their synthesis represents less of a metabolic burden to the cell.

We then proceeded to investigate whether the landscape of interactions between the genes of the HFS were similar to or different from those of the global genetic interaction network. We observed that the incorrect predictions of gene essentiality led to further inaccuracies in the prediction of synthetic lethal (SL) pairs. Only 2 of the 163 predictions of SL pairs in the network were experimentally verified leaving the specificity of the HFS at 8.00 %, which was slightly lower than that of CN (10.51 %). As might have been predicted, although the essential genes themselves were under-represented in the HFS, there was an enrichment of SL interactions among the genes in the HFS network in comparison to that of the global genetic interaction network (0.08 vs. 2.53 %). This is congruent with the idea that the presence of many isozymes in this vital subset of the central metabolic network results in an under-representation of essential enzymes, but a high proportion of synthetically lethal interaction between pairs of genes encoding the enzymes of the HFS. The false-negative interactions were predicted among 46 genes that encode components of the electron transport chain complexes I, III and IV. In contrast to the case of essential genes, the SL pairs were identified to be over-represented in the sub-network. A recent study on the characterization of genetic interaction networks in yeast metabolism also reported a negative correlation between single-gene deletant fitness in FBA predictions and the FBA predicted epistasis (Szappanos et al. 2011). The 23 false-positive interactions in HFS were predicted among 30 genes whose annotations are significantly enriched for the GO Process terms ‘generation of precursor metabolites and energy’ (p = 5.21 × 10^−13^), and ‘programmed cell death’ (p = 5.17 × 10^−4^); see Table 1 and ESM8.

### Stoichiometric model of the yeast metabolic network indicates a high energy burden associated with generating biomass

The electron transport chain in *S. cerevisiae* serves as the major route of ATP production for cells grown on relatively low concentrations of glucose. Cellular proliferation, which is associated with biomass production in metabolic models, is the major energy-consuming process carried out by the cells. Production of biomass, with its high metabolic burden in terms of energy requirements, serves as an ideal platform for the investigation of how sensitive the electron transport chain (one of the weakest links in the yeast metabolic model) would be to changes in the chemical composition of biomass (as represented in the model) as well as to macronutrient limitation constraints introduced to the model. For this purpose, the stoichiometric coefficients of the 36 biomass constituents were individually varied by an arbitrarily selected factor of two under conditions of nutrient sufficiency as well as under conditions of limitation for one of four major macronutrients required for the growth and proliferation of *S. cerevisiae* in a chemically-defined nutrient environment: glucose, ammonium, phosphate and sulphate as primary sources of carbon, nitrogen, phosphorus and sulphur, respectively (ESM9, ESM10 and ESM11). The empirical data that are available on the biomass composition of *S. cerevisiae* indicated that the previously reported values for the biomass constituents varied within a 10-fold range of the values that were implemented in Yeast 7.00 model. The only exception to this is the lipids, for which even consensus molecular weights are still unavailable (Albers et al. 1996; Bruinenberg et al. 1983; Henry 1982; Oura 1972; Schulze 1995; Vaughan-Martini and Martini 1993). Therefore, the artificially created variations in the biomass composition, which remained within a fourfold range, still yielded results falling within the possible solution space, whose actual limits were set by the available empirical data. For convenience, we refer to the original biomass composition as the “standard biomass configuration (SBC)” and the 72 in silico generated yeast cell configurations with altered biomass composition as “biomass-reconfigurations (BR)” from this point onwards.

**Fig. 1:**
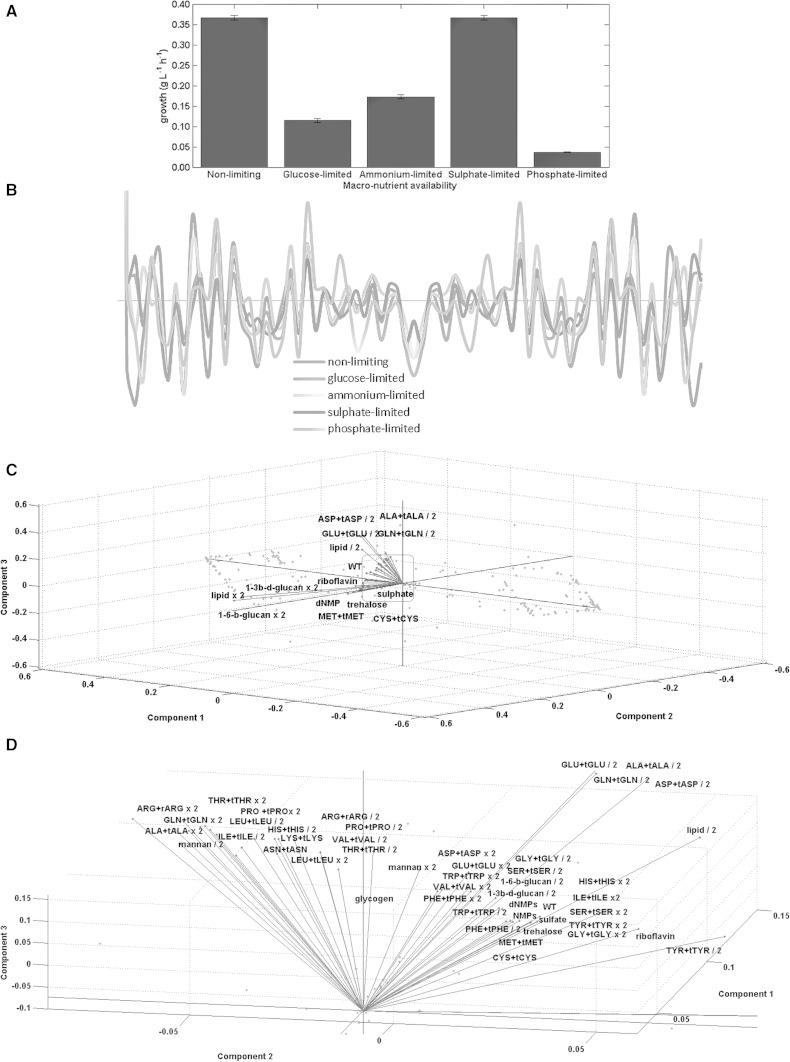
Variation in fluxes and the principal components analysis of the flux distributions in response to changes in biomass composition. Variation in growth-rate predictions under different nutritional limitations and under non-limiting conditions. *Error bars* indicate the variance observed in each dataset as a result of the biomass reconfigurations (**a**). The number of reactions whose fluxes remain unchanged with respect to the standard biomass configuration under different conditions of nutrient availability is presented as the ‘real’ components of the Fourier transform (**b**). The different biomass reconfigurations were anonymously represented as a continuous array of variations along the abscissa. The bi-plots of the loading vectors (in *blue*) and the factor scores (in *red*) represent the variation in biomass composition and the simulated reaction flux, respectively. The three principal components; Component 1, Component 2 and Component 3 cover 84, 4, and 3 % of the variance in the data, respectively (**c**). The plot in (**d**) is a zoomed-in perspective of the region shown in the *red rounded rectangle* in (**c**) (Color figure online)

Nearly half (49.7 %) of the reactions in the metabolic network were affected by changing the biomass content under different conditions of nutrient availability. Growth rates were determined to be similar under different metabolic reconfigurations for the same environmental condition. Sulphate limitation did not impair growth as predicted by the model. However, growth rates were lower for the case of the three remaining limitations; those of ammonium, glucose and phosphorus, with the highest growth impairment observed under phosphorus limitation and the lowest under ammonium limitation (Fig. 1a). A previous study on the adaptation of *S. cerevisiae* cells to growth in nutrient-limited chemostats reported much more constrained genotypic and phenotypic outcomes for sulphate-limited populations in contrast to populations grown under glucose- or phosphate-limitation (Gresham et al. 2008). Several fluxes were affected only under a sub-set of conditions with ca. 15–39 % of the total number of reaction fluxes remaining unchanged across different BR. Although this value was dependent on the environmental condition under investigation; numerically, the overall trend across the SBC and the BR remained similar (Fig. 1b).

We explored how different biomass reconfigurations affected the in silico distribution of fluxes under non-limiting nutrient conditions and under limited macronutrient availability. More reactions were affected by varying the lipid content of biomass under non-limiting conditions and under sulphate or phosphate limitation. Variations in 1-3 β-d-glucan and glutamate content resulted in variations in a greater number of fluxes under glucose and ammonium limitations, respectively. On the other hand, fewer reactions were affected by variations in trehalose content (non-limiting and glucose-limited environments), cysteine content (ammonium- and phosphate-limited environment) and tryptophan content (sulphate-limited environment) in the metabolic network. More reactions were significantly affected by increasing (as compared to decreasing) the structural/storage carbohydrate content of biomass under non-limiting conditions, glucose, or ammonium limitation (p value: <0.01, <0.01, and <0.05, respectively); the dNMP content of biomass under non-limiting conditions, glucose or sulphate limitation (p value: <0.02, <0.05, and <0.02, respectively); or the NMP content of biomass under glucose or phosphate limitation (p value: <0.01 and <0.05, respectively).

We carried out an orthogonal transformation of the flux distributions as a function of different BR and the first 3 principal components were sufficient to capture more than 91 % of the total variation in the dataset. The weights of structural constituents of biomass such as 1-3-β-glucan and 1-6-β-glucan and the lipid content along with alanine, aspartate, glutamate, and glutamine were the highest among the biomass constituents (Fig. 1c). The large contribution of the structural constituents of biomass on the scores was expected since the cell wall constituents comprise more than 30 % of the total biomass. The contributions of the NMP and dNMP variables were similar to that of the standard configuration, whereas a variation in amino acid composition resulted in a diverse range of responses (Fig. 1d). The biomass components causing the highest variation in the data were the lipid component, 1-3 β-d-glucan, 1-6 β-d-glucan, alanine, aspartate, glutamate and glutamine. Consequently, the highest variation, indicated by the flux scores, was observed in the fluxes through transport and other reactions involved in lipid and sphingolipid biosynthetic routes in accordance with our earlier findings.

**Fig. 2:**
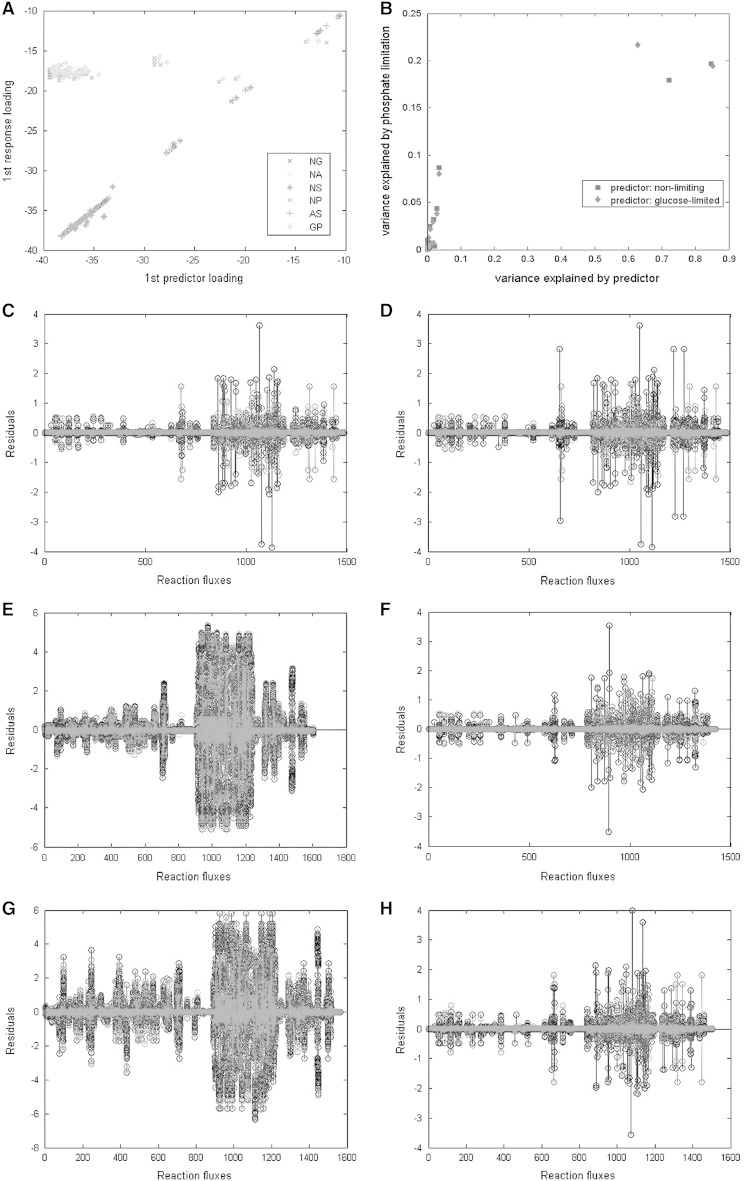
Partial least-squares (PLS) regression analysis of flux distributions under different conditions of nutrient availability and the plot of residuals on the non-zero response fluxes for each reaction. The plot of the first response loading against the first predictor loading for the dataset pairs under investigation is displayed. The first initial in the legend denotes the predictor dataset and the second initial denotes the response dataset, with the following abbreviations; *N* non-limiting, *G* glucose-limited, *A* ammonium-limited, *S* sulphate-limited, and *P* phosphate-limited (**a**). The relative variance explained in phosphate limitation by that of the predictor, in either non-limiting or glucose-limited conditions, is given in (**b**). The residuals were plotted for each reaction that displays a response across the dataset pairs under investigation; NG (**c**), NA (**d**), NP (**e**), NS (**f**), GP (**g**), and AS (**h**) as a measure of the deviation of the simulation from the response predicted by the PLS regression. The first initial denotes the predictor dataset and the second initial denotes the response dataset with the following abbreviations; *N* non-limiting, *G* glucose-limited, *A* ammonium-limited, *S* sulphate-limited, and *P* phosphate-limited. Please note the difference in the y-axis scales between (**e**) and (**g**) and the remaining residual plots

We then proceeded to investigate whether these observations could be used as predictors to estimate the flux response of yeast under conditions of limitation for one of the macronutrients in the growth medium by employing PLSR. Thus, we used ammonium-limitation as a predictor for the landscape under sulphate-limitation (since both these macronutrients are used in amino acid metabolism). Glucose-limitation was used as a template for predicting the distribution of fluxes in response to phosphate-limitation owing to the tightly interwoven roles of these two macronutrients in energy metabolism. The landscape under non-limiting conditions was observed to effectively predict the response observed under glucose, ammonium or sulphate limitation with just the first loading predictor and response loadings explaining more than 80 % of the total variance among the datasets. Similarly, the distribution of fluxes under ammonium-limitation could successfully be utilized as a predictor of the response under sulphate-limitation (Fig. 2a). On the other hand, the set of fluxes simulated under the non-limiting or glucose-limited in silico environments failed to predict the response of yeast under phosphate-limitation, with less than 25 % of the total variance under phosphate-limitation being explained by the predictors (Fig. 2b). The metabolic reconfiguration of the cell in response to variations in its biomass content under non-limiting environmental conditions served as an adequate template for predicting how flux changes under glucose, ammonium or sulphate limitations, but not under phosphate limitation. The availability of phosphate in sufficient concentrations, being a major parameter in energy-linked processes, perhaps necessitated a novel fluxomic rewiring of the metabolic network whereas the limitation of any one of glucose, ammonium or sulphate simply reduced the rate of metabolic activity while maintaining a similar metabolic landscape to that observed under conditions in which those nutrients were non-limiting.

The residual profiles across all reactions in the yeast metabolic network were observed to display similar trends with the only difference being that the magnitude and residual values in the response fluxes under phosphate limitation were higher than in any of the remaining cases under investigation (Fig. 2c–h). Specifically, the reactions for which the predicted fluxes had high residuals were significantly enriched for lipid metabolic processes (p value <5E−13) indicating a possible inadequacy in the model’s representation of lipid metabolism.

**Fig. 3:**
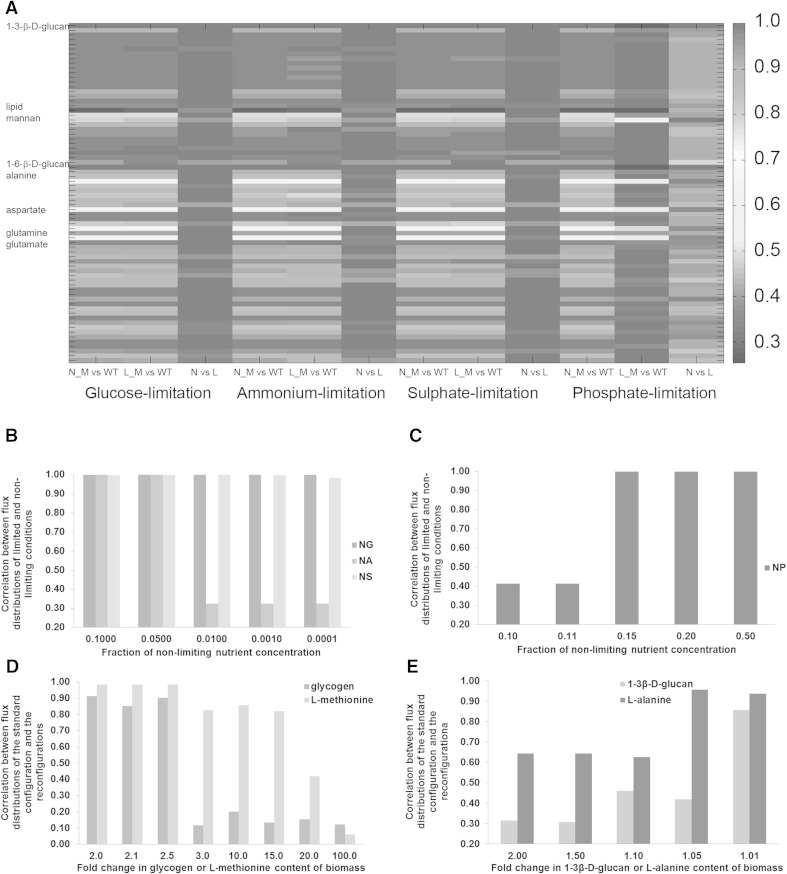
Heat map of the correlations among the flux distributions and the sensitivity analysis associated with the highly correlated and non-correlated fluxes. The correlations among the flux distributions for 72 biomass-reconfigurations, each represented by a single row in the map, were summarized for each macronutrient limitation, represented by tri-columns. For each macronutrient limitation, three sets of correlation analyses were carried out; between the different biomass configurations (M) and the standard configuration (WT) under non-limiting (N) and nutrient-limited (L) conditions, as well as between non-limiting (N) and nutrient-limited (L) conditions for each biomass-reconfiguration (**a**). The variation in the correlation between fluxes as a response to the severity of the change in limitation or biomass reconfiguration is displayed in the following plots. The variation in the correlation between the flux distributions of the standard biomass configuration simulated for growth under non-limiting and under either one of glucose, ammonium, or sulphate limitations is given in (**b**) and that for growth under non-limiting and under phosphate limitation is given in (**c**). The variation in the correlation between the flux distribution of the standard biomass configuration and of the reconfigurations achieved by increasing the glycogen or methionine content of biomass is given in (**d**). The variation in the correlation between the flux distributions of the standard biomass configuration and of the reconfigurations achieved by increasing the 1-3 β-d-glucan content or decreasing the l-alanine content of biomass is given in (**e**)

The flux distributions for both the SBC and the BR were highly positively correlated (PCC > 0.85) between non-limiting and glucose-, ammonium-, or sulphate-limited conditions; whereas they were uncorrelated (PCC < 0.5) between the non-limiting and the phosphate-limited environments, except for increasing the structural content of biomass through 1-3 β-d-glucan, 1-6 β-d-glucan and the lipid content (PCC > 0.9). The statistical dependence of the flux distributions calculated for the BR, in which the 1-3 β-d-glucan, 1-6 β-d-glucan, mannan, or lipid content of the biomass was increased and lipid, ALA, ARG, ASN, ASP, GLN, GLN, GLU, PRO or THR content of the biomass was reduced, was low between the nutrient non-limiting and limiting conditions independent of which macronutrient was supplied in growth-rate limiting amounts in the environment (Fig. 3a).

Although we could not observe any correlation between the impact of reconfiguration on the distribution of the fluxes and either the fraction of biomass that the reconfigured constituent represents or the connectivity of that constituent, a striking feature was observed in the case of the lipids. Although constituting a relatively small fraction of the total biomass (0.24 %; as described in Y7.00), they are involved in a substantial portion of the metabolic network (1471 reactions, 42 % of all enzymic and transport reactions included in the model) and it is likely that this high connectivity was the reason behind the large impact of reconfigurations that we observed when changing the lipid content of the biomass. Due to the highly connected structure of metabolic networks, a single perturbation was previously reported to necessitate the adaptation of the network to the new state as a whole (Wagner and Fell 2001). Therefore, it is not surprising that the highly connected lipid metabolism was a major contributor of the variation we have observed in the distribution of fluxes under the stated conditions. In conjunction with the notion that it is the high connectivity of the lipid metabolism with the remaining parts of the metabolism rather than the actual lipid content of the biomass, Nookaew and co-workers reported the lipid content of the biomass to have only a minor impact on growth under aerobic growth conditions, similar to those discussed here (Nookaew et al. 2008). Data presented and cited in Nookaew et al. (2008) indicate that lipids represent at least 2 % of yeast biomass, and this low lipid content of the Yeast X metabolic models in comparison to the previously available models such as iFF708, iLL672, iMM904, iND750, and iNN800 has been discussed by Aung et al. (2013). Although the predictive capability of metabolic models have improved considerably over the years despite the very low and unrealistic lipid content of the biomass, it does not circumvent the fact that these correct predictions on growth phenotype are usually achieved by an unrealistic distribution of the fluxes across the metabolic network.

### Flux distributions are sensitive to changes in variations in the biomass composition

The analysis presented above highlighted the fact that there is a very strong correlation in the distribution of the fluxes between most pairwise combinations of conditions (either nutrient limitations or biomass reconfigurations) investigated. In only a minority of such pairs, moderate to low correlations were found. Since this analysis was carried out using an arbitrarily selected twofold perturbation of the biomass composition between the selected conditions, the immediate questions arising from this observation were (i) whether the distribution of the fluxes would be affected by a sharp or a gradual perturbation in a component of the biomass and (ii) whether the wiring of the intracellular fluxes would remain unaffected by the magnitude of the change imposed under any of the physiological conditions or components of the biomass investigated. Either of these would indicate how sensitive the changes in the distribution of fluxes are to variations in biomass composition or the physiological status. We therefore performed a sensitivity analysis to evaluate the robustness of the differences and similarities identified in the present findings. Simulation of even very extreme conditions of limited macronutrient availability (near-starvation conditions created by reducing the respective nutrient uptake rates to 1/10000th of their original values) did not disturb the high correlation between flux distributions of cells with the SBC grown under non-limiting environmental conditions, or under glucose or sulphate limitation. On the other hand, the predicted flux distributions soon became uncorrelated as the ammonium available for uptake was reduced from 2 to 1 % of its non-limiting value. The decrease observed in the correlation was a sharp response rather than a gradual one (Fig. 3b). Conversely, we reduced the severity of phosphate limitation to determine the concentration beyond which growth under phosphate limitation and in non-limiting conditions become comparable. The correlation between these two flux distributions suddenly increased at a nutrient limitation threshold of 11 % and further increasing the concentration of phosphate up to 15 % of its non-limiting value resulted in a near-perfect correlation between the flux distributions (PCC > 0.99) (Fig. 3c).

This analysis of the robustness of the correlations within the dataset revealed that under, sulphate or glucose limitation, the calculated fluxes were shown to remain highly correlated with those of non-limiting nutrient availability, regardless of the severity of the limitation imposed on metabolism. On the other hand, a highly sensitive threshold of nutrient limitation was shown to exist for ammonium and phosphate, beyond which the distributions of fluxes were either highly correlated or non-correlated with those of the non-limiting conditions. Such a sensitive threshold was also determined to exist for varying the composition of biomass components in a similar evaluation. l-methionine, l-alanine, glycogen, and 1-3 β-d-glucan were investigated for this purpose. Glycogen (10.86 %) and 1-3 β-d-glucan (17.98 %) are among the most abundant constituents of biomass whereas l-methionine (0.24 %) and l-alanine (1.28 %) make markedly small contributions to the total cell mass. Increasing the l-methionine or glycogen content of biomass did not affect the overall flux distribution appreciably, whereas increasing the 1-3 β-d-glucan content of biomass or decreasing the l-alanine content resulted in flux distributions that were non-correlated with those of the flux predictions based on the SBC. A more severe alteration, by further increasing the glycogen content of biomass by 50 %, caused the distribution of fluxes to become non-correlated with that of the SBC (Fig. 3d). On the other hand, a relatively high correlation (PCC > 0.85) between the flux distribution of the SBC and those of the 1-3 β-d-glucan-reconfiguration and the l-alanine-reconfiguration could only be achieved by making any reconfiguration of biomass composition differ very little to the standard configuration, and changing the biomass content only incrementally (by <1 % and <5 % for the 1-3 β-d-glucan-reconfiguration and the l-alanine-reconfiguration, respectively) (Fig. 3e). This indicated that the metabolic network was very sensitive to changes in these major biomass components, but was rather robust to other changes, as we have demonstrated in the earlier case.

## Concluding remarks

Inaccurate description of medium and biomass composition is a source of false predictions of gene/enzyme essentiality in yeast metabolic models; a previous study suggesting that errors in the specification of biomass composition account for >30 % of the false predictions involving essential genes (Duarte et al. 2004). We have identified a link between energy-generating pathways and the identity of the growth-limiting nutrient with changes in biomass composition. The representation of phosphate limitation in flux simulations was observed to be particularly problematic, indicating that the accurate representation of phosphate metabolism was key to accurately modelling the metabolic network as well as to its definition of biomass. However, it should be noted that the high-flux sub-network was defined using experimental analyses where cells were grown on a complex, but chemically defined, medium containing a high glucose concentration, such that respiratory growth was repressed, even though oxygen was present. It is clear from our in silico analyses that a change in these physiological conditions might alter the high-flux sub-set but, nonetheless, highlight the importance of representing biomass composition in a condition-specific manner. All of this emphasises that more, and more accurate, empirical studies of the biochemical constitution of yeast biomass are essential if the predictive power of the yeast metabolic model is to be improved.

## Electronic supplementary material

Below is the link to the electronic supplementary material.

Supplementary material 1 (ZIP 11071 kb)
